# Profiles of distress related to sexual function, psychological distress, and sexual pleasure: a person-centered analysis of early maladaptive schemas and psychological inflexibility

**DOI:** 10.1093/sexmed/qfag062

**Published:** 2026-07-13

**Authors:** Valentim P Santos, Pedro J Rosa, Magda S Roberto, Andreia A Manão, Cátia Oliveira, Nuno Tomada, Joachim Osur, Diogo Telles-Correia, Patrícia M Pascoal

**Affiliations:** Faculdade de Medicina, Universidade de Lisboa, 1649-028, Lisboa, Portugal; HEI-Lab: Digital Human-Environment Interaction Labs, Lusófona University, 1749-024 Lisboa, Portugal; Instituto Superior Manuel Teixeira Gomes (ISMAT), 8500-656, Portimão, Portugal; CICPSI, Faculdade de Psicologia, Universidade de Lisboa, Alameda da Universidade, 1649-013, Lisboa, Portugal; HEI-Lab: Digital Human-Environment Interaction Labs, Lusófona University, 1749-024 Lisboa, Portugal; HEI-Lab: Digital Human-Environment Interaction Labs, Lusófona University, 1749-024 Lisboa, Portugal; Andrology and Sexual Medicine Unit, Urology department of Hospital da Luz Arrábida, 4400-346 Vila Nova de Gaia, Portugal; Amref International University, Nairobi 00625, Kenya; CICPSI, Faculdade de Psicologia, Universidade de Lisboa, Alameda da Universidade, 1649-013, Lisboa, Portugal; Faculdade de Medicina, Clínica Universitária de Psiquiatria e Psicologia Médica, Universidade de Lisboa, 1649-028, Lisbon, Portugal; HEI-Lab: Digital Human-Environment Interaction Labs, Lusófona University, 1749-024 Lisboa, Portugal; Faculdade de Medicina, Clínica Universitária de Psiquiatria e Psicologia Médica, Universidade de Lisboa, 1649-028, Lisbon, Portugal; Faculdade de Medicina, PSYLAB, Instituto de Saúde Ambiental (ISAMB), Universidade de Lisboa, 1649-028, Lisbon, Portugal

**Keywords:** sexual function, sexual dysfunction, sexual distress, sexual pleasure, early maladaptive schemas, psychological inflexibility

## Abstract

**Background:**

Sexual distress and sexual pleasure are two clinically distinct parameters used to evaluate sexual dysfunction, alongside psychological distress. Various psychological processes and problems have been implicated in the etiology and maintenance of sexual dysfunction. Two transdiagnostic processes, Early Maladaptive Schemas (EMS) and Psychological Inflexibility (PI), have been associated with psychopathology. However, knowledge about their effects on sexual health outcomes, namely distress and pleasure, is lacking.

**Aim:**

This cross-sectional study investigates whether different profiles related to sexual dysfunction—defined by distress related to sexual function, sexual pleasure, and psychological distress—show distinct patterns of EMS and PI and how these patterns may inform targeted interventions.

**Methods:**

Data from 612 participants [*M*_age_ = 37.95 years, 77.0% identified as women (n = 471)] were included in our analysis. A latent profile analysis (LPA) was conducted to identify distinct profiles of distress related to sexual function, sexual pleasure, and psychological distress. Later, Bolck–Croon–Hagenaars procedures were employed to identify in-between profile differences across EMS and PI.

**Outcomes:**

The outcomes of our study were EMS and PI scores across profiles.

**Results:**

Our analysis yielded 3 distinct profiles: Low Distress/High Pleasure, High Distress/Low Pleasure, and Deeply Affected Pleasure profiles, with the latter presenting with greater symptom severity. Sexual pleasure is the indicator that best discriminates the latter profile from the former two. There is a significant increase in various EMS from the Low Distress/High Pleasure to the High Distress/Low Pleasure profile, specifically in those belonging to the domains of Disconnection and Rejection and Impaired Autonomy and Performance. The early maladaptive schema that differs in the Deeply Affected Pleasure profile (when comparing with the High Distress/Low Pleasure profile) is Emotional Deprivation. Psychological Inflexibility differs in these profiles and is more significant in the Deeply Affected Pleasure cluster.

**Clinical Implications:**

Sexual pleasure emerges as a relevant factor to consider in clinical evaluation and intervention. Also, targeting EMS and PI might prove beneficial in patients presenting with increased distress related to sexual function and/or diminished sexual pleasure.

**Strengths and Limitations:**

This article incorporates both negative (ie, sexual distress) and positive (ie, sexual pleasure) sexual outcomes as components of a sexual dysfunction and our results put sexual pleasure at the center of clinical conceptualization. Various limitations restrict our findings, namely, the characteristics of the sample (mostly highly educated women), the study design (cross-sectional), the non-inclusion of gender, sexual orientation and relational configuration diversity, and the non-inclusion of other determinants of sexual health (eg, relational factors, chronic disease).

**Conclusion:**

EMS and PI are associated with distress related to sexual function, sexual pleasure, and psychological distress, as per the profiles presented. Specifically, the Emotional Deprivation schema might be of importance in patients with diminished sexual pleasure.

## Introduction

Sexual Medicine experts have advocated for the use of a patient-centered framework in Sexual Medicine that acknowledges that sexual dysfunctions are self-reported clinical conditions framed by the experience of sexual distress related to sexual function and sexual pleasure.^1^^,^^2^ Sexual dysfunctions’ etiology and maintenance are carried out by interpersonal, psychological, and social problems,^3^ and are characterized by high levels of distress related to sexual function, which is an important motivator for people seeking treatment.^4^ Within the basic evaluation of sexual dysfunction, clinicians need to address patients’ psychosocial history, recognizing that diverse psychological (eg, psychopathology^5^ and their pharmacotherapy^6^) and interpersonal factors may be influencing the patient’s negative emotional experience.^3^^,^^7^

Early Maladaptive Schemas (EMS) are core structures that constitute the theoretical foundation of Schema Therapy (ST), an approach that expands on Cognitive-Behavioral Therapy (CBT) and that was developed to treat patients with personality disorders who were not being adequately managed with traditional CBT.^8^ Schema Therapy was developed by Jeffrey E. Young in a seminal work where he conceptualized and operationalized EMS as self-detrimental emotional and cognitive patterns that arise early in one’s development and extend into one’s adult life.^9^^,^^10^ Early Maladaptive Schemas develop when core emotional needs are unmet and, once established, play an important role in how an individual thinks and behaves, often leading them to paradoxically recreate these experiences in their adult lives—the perpetuation of schemas.^8^^,^^11^ Young identifies 18 EMS, grouped into 5 schema domains that are organized according to their underlying themes. A brief description of each EMS and schema domain is present in Table 1.

**Table 1:** Schema domains and EMS; adapted from Young et al.^8^

**Schema domain**	**Early maladaptive schema**	**Brief description**
**Disconnection and Rejection** (ie, the expectation that one’s core emotional needs will not be met in a predictable manner)	Abandonment/instability	The expectation that attachment figures are unreliable, inconsistent, or likely to withdraw support
	Mistrust/abuse	Seeing others as malevolent, leading to anticipatory expectations of exploitation, harm, or humiliation
	Emotional deprivation	Belief that needs for emotional nurturance, empathy, and protection will not be adequately met
	Defectiveness/shame	Internalized conviction of being fundamentally defective, inferior, or unworthy of love and respect
	Social isolation/alienation	Stable perception of being disconnected from, and qualitatively different than, any social groups
**Impaired Autonomy and Performance** (ie, difficulties in developing a sense of independence and competence)	Dependence/incompetence	Belief that one lacks competence to manage everyday responsibilities without substantial external guidance
	Vulnerability to harm or illness	Persistent overestimation of imminent catastrophic events (eg, medical, financial)
	Enmeshment/undeveloped self	Diffuse self-definition characterized by over-involvement with significant others and impaired autonomy
	Failure	Globalized expectations of failure and underachievement relative to age- and context-appropriate standards
**Impaired Limits** (ie, difficulties respecting boundaries or exercising self-control)	Entitlement/grandiosity	Overevaluation of one’s importance with expectations of special rights and exemption from normative limits
	Insufficient self-control/self-discipline	Chronic difficulty inhibiting impulses or tolerating distress in pursuit of long-term goals
**Other-Directedness** (ie, focusing excessively on meeting the needs and expectations of others at the expense of their own)	Subjugation	Habitual suppression of personal needs and preferences due to anticipated retaliation or rejection
	Self-sacrifice	Excessive prioritization of others’ needs driven by guilt, responsibility, or fear of harming others
	Approval-seeking/recognition-seeking	Self-evaluation heavily contingent on external validation and social approval cues
**Overvigilance and Inhibition** (ie, suppressing emotions, impulses, and spontaneity to meet internalized rules and standards)	Negativity/pessimism	Cognitive bias toward emphasizing potential loss, error, and adversity over positive contingencies
	Emotional inhibition	Rigid suppression of emotional expression and spontaneity to avoid disapproval or perceived loss of control
	Unrelenting standards/hypercriticalness	Internalization of excessively high-performance standards associated with self-criticism and reduced satisfaction
	Punitiveness	Tendency to advocate harsh punishment rather than compassion for self or others after mistakes or norm violations

Early Maladaptive Schemas and psychopathology have been consistently associated. Individuals with a clinical diagnosis of depression^12–15^ and anxiety disorders^14–16^ show increased levels of EMS, and interventions that aim to reduce the impact of EMS on these patients have been linked to a reduction in symptom severity.^17^^,^^18^ These associations seem to be consistent in non-clinical samples as well, where individuals reporting higher psychological distress present with higher scores of EMS,^13^^,^^16^ and support that EMS are linked to the etiology and maintenance of emotional problems. Complementarily, certain processes have been identified as stemming from EMS and may explain their role in emotional problems. For example, Fischer et al. opened a research venue where they proposed a model where Psychological Inflexibility (PI) fully mediated the effects of EMS on psychopathology.^19^

PI reflects a rigid tendency for behavior to be dominated by emotional (eg, anxiety, shame) and cognitive (eg, self-criticalness, catastrophizing) reactions, rather than guided by valued goals and contextual contingencies, and it is the treatment target in Acceptance and Commitment Therapy (ACT), a third-wave CBT.^20^ Research supports PI as a core transdiagnostic process underlying several forms of psychiatric conditions (eg, depression and anxiety disorders)^21^ and ACT protocols have efficaciously reduced symptom severity in these patients.^22^^,^^23^

Despite the established interrelation between sexual distress and psychopathology, namely, as part of the internalized spectrum of emotional problems,^24^ the extent to which EMS and PI, two transdiagnostic mechanistic processes,^14^^,^^21^ act on sexual dysfunction remains understudied. There is some preliminary evidence that EMS are associated with poorer sexual health outcomes. Two cross-sectional studies with mostly community samples aimed to explore the association between EMS and sexual function.^25^^,^^26^ They found that the domain of Impaired Autonomy and Performance (when controlling for sociodemographic variables and psychopathology) was negatively correlated with sexual function in both men^25^ and women.^26^ However, they used clinical samples with a low number (eg, clinical sample n = 24^26^), studied men’s and women’s sexual function separately and with non-comparable measures, did not consider the experience of sexual pleasure, and sexual distress was not directly measured.

Additionally, Nobre proposed a cognitive–emotional model and developed a study where maladaptive beliefs and specific sexual cognitive schemas activated in sexual contexts in men (n = 49 with a clinical diagnosis of sexual dysfunction) trigger dysfunctional thoughts during sexual activity, resulting in sexual impairment.^27^ Highlighting the central role of sexual schemas in shaping sexual function and suggesting that sexuality-specific cognitive structures may influence sexual outcomes. The authors assessed the role of sexual schemas but did not address the role of EMS, which operate across multiple life domains and may also be relevant in sexual contexts. In addition, existing evidence suggests that certain EMS (eg, defectiveness/shame and emotional deprivation) negatively predict sexual self-confidence and sexual adjustment.^28^ However, both of these studies failed to include the experience of distress and pleasure. Therefore, the associations between EMS and sexual dysfunction, though promising, need to be more nuanced and clarified in terms of their clinical relevance.

PI has also been associated with sexual dysfunction. For instance, Saito et al. showed that men with erectile dysfunction younger than 40 years have higher PI and might be eligible for ACT^29^; however, despite measuring psychopathology, they focused solely on male sexual function and did not measure the level of sexual distress or sexual pleasure experienced. As such, the results need to be expanded to address other key characteristics of sexual dysfunctions (eg, sexual pleasure, sexual distress) and take a more nuanced approach to the role of PI.

It is important to note that, while PI may emerge following the activation of EMS in relevant contexts, the two constructs are theoretically distinguishable, operating at different levels of analysis. Furthermore, PI entails additional mechanisms (eg, cognitive fusion, experiential avoidance).^30^^,^^31^ Subsequent categorization of how these mechanisms relate to EMS activation in psychopathology is warranted. Even though there are findings^19^ in which PI statistically mediate the relationship between EMS and psychological outcomes, such evidence does not permit inferences regarding causal mediation due to the cross-sectional nature of the evidence. As such, the observed effects presented should be interpreted with caution, and as model-consistent associations rather than definitive evidence of a mediating process.

Considering that both EMS and PI are related constructs that account for negative and positive individual and interpersonal mental-health-related outcomes, we aim to expand existing knowledge in the field by understanding how latent profiles distinguished by core characteristic criterion of sexual dysfunctions differ across EMS and PI. Specifically, our research question is: What latent profiles of Distress Related to Sexual Function (DSF),^32^ Sexual Pleasure, and Psychological Distress can be identified, and how do the levels of EMS and PI differ across them?

## Methods

### Participants

Power analysis in latent profile analysis (LPA) remains an emerging area, as there are currently no formulas or definitive criteria for determining the required minimum sample size. Sample size requirements depend on several factors (eg, number of latent profiles and the degree of separation between them), which are typically unknown *a priori*. Yet, in the present study, we followed recommendations derived from simulation research by adopting a minimum sample size of 500 participants, as suggested by Finch and Bronk.^33^

This study is based on a convenience sample of 673 participants. Inclusion criteria were being 18 years old or older, being able to understand the Portuguese written language, and having had sexual activity in the last 6 months. Sexual activity was defined as “… mutual stimulation of genitals, oral sex, anal sex, intercourse, and other forms of face-to-face sexual stimulation.”^34^ Forty-nine participants were excluded due to leaving at least 1 item from each scale unanswered, resulting in a total of 624 participants.

The sociodemographic characteristics of the sample are presented in Table 2.

**Table 2:** Sociodemographic characteristics of the sample.

**Gender**	**N (%)**	**Sexual orientation**	**N (%)**	**Relational configuration**	**N (%)**
Woman	471 (75.5)	Heterosexual	501 (80.3)	Without relationships	105 (16.8)
Men	141 (22.6)	Lesbian	17 (2.7)	Monogamous	459 (73.6)
Non-binary	8 (1.3)	Gay	27 (4.3)	Consensual non-monogamous	21 (3.4)
“I would rather not answer”	2 (0.3)	Bisexual	44 (7.1)	Sporadic relationships	31 (5.0)
Other	2 (0.3)	Queer	9 (1.4)	Other	8 (1.3)
		Without category/“label”	11 (1.8)		
		Questioning	8 (1.3)		
		Other	7 (1.1)		
**Educational level**	**N (%)**				
Without higher education (≤ 12 years)	192 (30.8)				
With higher education (> 12 years)	432 (69.2)				

### Measures

#### Sociodemographic characteristics

A brief questionnaire was developed to identify the sociodemographic characteristics of our sample, including age, gender, sexual orientation, and relational configuration.

#### Young Schema Questionnaire–Standardized Items 3rd version

The Young Schema Questionnaire–Standardized Items 3rd version (YSQ-S3)^35^ is a 90-item questionnaire used to evaluate the 18 EMS as theoretically proposed by Young. Each item is rated on a 6-point Likert scale from 1 (“completely untrue of me”) to 6 (“describes me perfectly”) and the results summed, for a total of 5-30 points for each schema. This scale has been translated and validated for use in the Portuguese population.^36^ In our study, internal consistency was acceptable for all EMS (α = 0.71-0.91) except for the Entitlement/Grandiosity schema (α = 0.64).

#### Kessler Psychological Distress Scale of 6 items

The Kessler Psychological Distress Scale of 6 items (K6)^37^ consists of 6 items assessing the frequency of non-specific symptomatology associated with psychological distress over the past 30 days. Each item is rated on a 5-point Likert scale from 0 (“none of the time”) to 4 (“all of the time”), and the results are summed. This scale came from the 10-item version that has been adapted and validated for use in the Portuguese population.^38^ In this study, this scale showed good internal consistency (α = .87).

#### Acceptance and Action Questionnaire—Second Version

The Acceptance and Action Questionnaire—Second Version (AAQ-II)^39^ is originally a 10-item scale that measures the level of PI experienced by a subject; subsequent exploratory and confirmatory analyses led to a final version of the questionnaire with 7 items. Each item is rated on a 7-point Likert scale from 1 (“never true”) to 7 (“always true”) and the results are summed. This scale has been adapted and validated for use in the Portuguese population^40^ and subsequent exploratory and confirmatory analysis established a final version of the questionnaire with 7 items. In our study, the AAQ-II 7 item version showed excellent internal consistency (α = .91).

#### Sexual Function Evaluation Questionnaire—Problem Distress subscale

The Sexual Function Evaluation Questionnaire (SFEQ)^41^ is a 16-item questionnaire designed to measure overall sexual function; currently, the Portuguese version is under validation. In this study, we use the Problem Distress subscale to assess DSF, consisting of 8 items rated on a 5-point Likert scale [eg, from 0 (“Not a problem”) or 1 ("Not at all distressed") to 4 (“Very distressed”)]. The Problem Distress subscale showed acceptable internal consistency (α = 0.77).

#### Sexual Pleasure Scale

The SPS^42^ is a set of 3 items that aims to evaluate the extent of someone’s sexual pleasure obtained through sexual relationships, sexual activities, and sexual intimacy, respectively. The respondents answer on a 7-point Likert scale from 1 (“not pleasurable at all”) to 7 (“very pleasurable”), and the results are summed. In our study, the SPS showed good internal consistency (α = 0.80).

### Procedures

This study is part of a larger mixed-methods project about sexual distress funded by the European Society for Sexual Medicine (RG 21-01). We used participatory design techniques^43^ in collaboration with Musex (https://musex.pt), Associação Gerador, and certified sex therapists who helped to refine the content of the survey, tested, and disseminated it in different social media after ethical approval (CEDIC-2022-15-7). It was collaboratively decided to use online recruitment and data collection to make the survey more inclusive and maximize its outreach.^44^ There were open questions (eg, profession) and reversed questions to detect non-attentive participants whose answers were deleted. Geolocation information and IP addresses were deleted, and the database was protected with a password known only to team members. The informed consent page mentioned that the dataset would not be shared. The contacts of the main researcher and a list of public services were available for those people who wanted to autonomously look for help. Data were collected between March 2023 and October 2024.

### Statistical analysis

People who identified as non-binary (n = 12) were excluded due to under-representativeness in the sample which compromises statistical analysis, resulting in 612 participants. Due to the highly unbalanced distribution of participants across sexual orientation and relationship categories, no further analyses involving these variables were conducted.

Firstly, we assessed the degree of conceptual and empirical overlap between EMS and PI, and evaluated discriminant validity using the heterotrait–monotrait ratio of correlations (HTMT) within a structural equation modeling framework. Values below .85 indicate adequate construct distinctiveness.^45^ Subsequently, we examined the bivariate association between these variables using correlation analysis. EMS was operationalized as the average rating across items to better approximate the model presented by Fischer et al.^19^ The variance inflation factor (VIF) and tolerance, used as measures of multicollinearity, were also computed for both the EMS and PI mean composite scores.

Secondly, a descriptive analysis of the indicators used in LPA was performed (Table 4). Three indicators of Sexual Dysfunction were used: DSF, Sexual Pleasure, and Psychological Distress. Current diagnostic frameworks (eg, DSM-5-TR^1^) conceptualize sexual dysfunction as the presence of self-reported alterations in sexual response and the presence of clinically significant distress and/or diminished sexual pleasure. The combination of indicators chosen allows for the identification of distinct phenotypical profiles, with different levels of impairment and distress. Furthermore, the inclusion of both sexual-specific (ie, sexual distress) and general psychological distress acknowledges the broader psychological context in which sexual difficulties occur, as suggested by the self-identification of distressing emotional experiences by individuals with distressing sexual experiences.^32^^,^^46^ Sexual pleasure was included as a positive outcome of sexual activity.^2^ Although other factors mentioned (eg, interpersonal factors) are undoubtedly relevant in sexuality, they were not conceptualized as manifestations of individual psychological problems related to sexual difficulties, and were, therefore, not included as profile indicators. This decision was also made to preserve conceptual clarity and parsimony in the LPA.

Then we ran a series of sequential models with 1-6 profiles to identify the best-fitting solution. The optimal number of profiles was determined using a combination of statistical and theoretical criteria^47^: (1) the entropy value >0.9, indicating high classification accuracy (the higher, the better); (2) the Akaike information criterion (AIC), Bayesian information criterion (BIC), Adjusted Bayesian information criterion (ABIC), where models with a lower value representing a better fit; (3) a non-significant Vuong–Lo–Mendell–Rubin likelihood ratio test^48^^,^^49^ indicated that the *K*-class model did not provide a significantly better fit than the (*K* – 1)-class model; and (4) parsimony and interpretability, with profiles containing fewer than 5% of the sample excluded from consideration.^50^

Participants were then assigned to their most likely profile based on the latent profile posterior distribution. Profile-specific differences in EMS and PI were tested using automatic Bolck–Croon–Hagenaars procedures^51^ with omnibus Wald chi-square tests of equality across profiles.^47^ Bonferroni adjustment was applied for pairwise comparisons. Effect sizes for pairwise comparisons were estimated using Cohen’s *d* and were interpreted using the conventional benchmarks of 0.20/0.50/0.80 (small, medium, and large, respectively).

IBM SPSS Statistics (Version 27) was used for preprocessing, descriptive and correlational analysis, and to assess multicollinearity (VIF and tolerance). LPA was conducted in Mplus (8.3), and the HTMT ratio was computed in JASP (0.95.4). The level of statistical significance was set at *P* < .05.

## Results

The HTMT ratio of correlations was 0.824 (<0.85), indicating that EMS and PI were conceptually distinct. Despite their strong correlation (*r* = 0.77), multicollinearity diagnostics (VIF = 2.47; tolerance = 0.41) suggested that the constructs were not redundant^52^ and could therefore be used separately in examining profile-specific differences.

Table 3 shows that the model fit improved for each added profile. Considering the models with an acceptable number of smallest class size (>5%; 2 and 3 profiles’ models), the model with 3 profiles had the best fit indices (the lowest AIC, BIC, and ABIC) and good entropy (0.907). Based on these indicators, the 3-profile model was selected as the optimal solution.

**Table 3:** Model fit indices and corresponding test of model change.

Number of classes	AIC	BIC	ABIC	Entropy	VLMR (*P*-value)	Smallest class size (%)
2	4931.979	4976.147	4944.399	0.911	<.001	12.3
**3**	**4782.063**	**4843.898**	**4799.450**	**0.907**	**<.001**	**5.3**
4	4703.014	4782.515	4725.369	0.910	<.01	3.1
5	4635.748	4732.916	4663.071	0.898	<.05	4.6
6	4583.140	4697.975	4615.430	0.912	<.01	1.3

Abbreviations: AIC, Akaike information criterion; BIC, Bayesian information criterion; VLMR, Vuong–Lo–Mendell–Rubin Likelihood Ratio Test.

**Figure 1:**
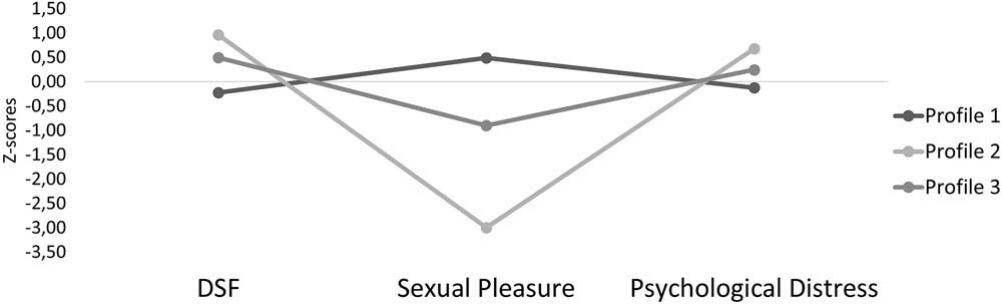
Z-standardized mean values of all LPA indicators for the final model.

The 3-profile solution comprised: Profile 1—the Low Distress/High Pleasure profile (n = 445; 72.71%); Profile 2—the Deeply Affected Pleasure profile (n = 32; 5.23%); and Profile 3 – the High Distress/Low Pleasure profile (n = 135; 22.06%). The profiles and the Z-standardized mean values for each LPA indicator are presented in Figure 1 and Table 4. Profiles 1 and 3 were labeled to reflect their relative levels of distress (psychological and DSF) and sexual pleasure based on their deviation from the mean average of these indicators. Profile 2 was labeled “Deeply Affected Pleasure” to highlight that sexual pleasure was the dimension that most strongly discriminated this profile from the others, showing markedly lower standardized scores of this indicator, associated with elevated DSF and psychological distress. All pairwise post hoc comparisons using the Bonferroni correction related to the indicators used in the LPA showed at least a medium effect size (Cohen’s *d* > 0.5) between profiles (Table 4).

**Table 4:** Z-standardized mean values of all LPA indicators and in-between profile differences.

**Measures**	**P1 (** $\boldsymbol{n}=\mathbf{445}$ **)** **Low Distress/High Pleasure *M* (SE)**	**P2 (** $\boldsymbol{n}=\mathbf{32}$ **)** **Deeply Affected Pleasure *M* (SE)**	**P3 (** $\boldsymbol{n}=\mathbf{135}$ **)** **High Distress/Low Pleasure *M* (SE)**	**Wald χ** ^**2**^ **(*P*-value)**	**Post hoc**	**Effect size**
DSF	−0.22 (0.05)	0.96 (0.23)	0.50 (0.12)	12.69 (<.01)	P1 < P2, P3	P1 vs P2: 1.18 P1 vs P3: 0.72
Sexual pleasure	0.49 (0.03)	−3.00 (0.16)	−0.90 (0.08)	87.40 (<.001)	P1 < P3 < P2	P1 vs P2: 3.49 P1 vs P3: 1.39 P2 vs P3: 2.10
Psychological distress	−0.12 (0.05)	0.68 (0.22)	0.25 (0.09)	5.18^ns^	—	—

Abbreviations: DSF, Distress Related to Sexual Function; M (SE), standardized means (standard errors) from LPA; ^ns^, non-significant; P, profile; post hoc, pairwise differences with Bonferroni adjustment.

Bolck–Croon–Hagenaars results, Bonferroni corrections, and effect sizes for sociodemographic variables, EMS and PI, are presented in Tables 5–7, respectively.

**Table 5:** Comparison of sociodemographic characteristics across latent profiles.

**Sociodemographic characteristics**	**P1 (** $\mathbf{n}=\mathbf{445}$ **)** **Low Distress/High Pleasure** $\%\left(\mathbf{n}\right)$	**P2 (** $\mathbf{n}=\mathbf{32}$ **)** **Deeply Affected Pleasure** $\%\left(\mathbf{n}\right)$	**P3 (** $\mathbf{n}=\mathbf{135}$ **)** **High Distress/Low Pleasure** $\%\left(\mathbf{n}\right)$	**Wald χ** ^**2**^ **(*P*-value)**	**Post hoc**	**Effect size**
**Gender**				7.18^ns^	—	—
Woman	76.6 (341)	90.6 (29)	74.8 (101)			
Man	23.4 (104)	9.4 (3)	25.2 (34)			
**Educational level**				1.73^ns^	—	—
Without higher education (≤12 years)	30.6 (136)	40.6 (13)	28.1 (38)			
With higher education (>12 years)	69.4 (309)	59.4 (19)	71.9 (97)			
	***M* (SE)**	***M* (SE)**	***M* (SE)**	**Wald χ** ^**2**^ **(*P*-value)**	**Post hoc**	**Effect size**
**Age**	37.43 (0.53)	45.39 (2.45)	37.92 (1.16)	10.15 (< .01)	P1, P3 < P2	P1 vs P2: 0.63 P2 vs P3: 0.55

Abbreviations: M (SE), means (standard errors); n, number of participants; ^ns^, non-significant; P, profile; post hoc, pairwise differences with Bonferroni adjustment.

**Table 6:** Comparison of EMS across latent profiles.

**Early maladaptive schemas** **(EMS)**	**P1 (** $\mathbf{n}=\mathbf{445}$ **)** **Low Distress/High Pleasure** ***M* (SE)**	**P2 (** $\mathbf{n}=\mathbf{32}$ **)** **Deeply Affected Pleasure** ***M* (SE)**	**P3 (** $\mathbf{n}=\mathbf{135}$ **)** **High Distress/Low Pleasure** ***M* (SE)**	**Wald χ** ^**2**^ **(*P*-value)**	**Post hoc**	**Effect size**
Abandonment/Instability	11.88 (0.29)	13.44 (1.26)	13.45 (0.61)	6.32 (<.05)	P1 < P3	P1 vs P3: 0.24
Mistrust/Abuse	12.12 (0.26)	14.65 (0.99)	13.84 (0.55)	12.38 (<.01)	P1 < P2, P3	P1 vs P2: 0.46 P1 vs P3: 0.29
Emotional Deprivation	9.01 (0.27)	15.78 (1.21)	12.30 (0.58)	35.01 (<.001)	P1 < P3 < P2	P1 vs. P2: 1.08 P1 vs P3: 0.53 P2 vs P3: 0.51
Defectiveness/Shame	7.67 (0.19)	12.14 (1.23)	10.32 (0.54)	31.80 (<.001)	P1 < P2, P3	P1 vs P2: 0.79 P1 vs P3: 0.50
Social Isolation/Alienation	12.80 (0.27)	16.38 (1.28)	14.97 (0.56)	17.66 (<.001)	P1 < P2, P3	P1 vs P2: 0.55 P1 vs P3: 0.35
Dependence/Incompetence	7.59 (0.15)	9.76 (0.79)	9.39 (0.41)	22.06 (<.001)	P1 < P2, P3	P1 vs P2: 0.56 P1 vs P3: 0.45
Vulnerability to Harm or Illness	12.17 (0.27)	13.56 (1.04)	13.59 (0.57)	5.92^ns^	—	—
Enmeshment/Undeveloped Self	8.69 (0.20)	10.55 (0.96)	9.86 (0.44)	8.45 (<.05)	P1 < P3	P1 vs P3: 0.25
Failure	8.68 (0.23)	10.91 (1.22)	10.88 (0.53)	15.97 (<.001)	P1 < P3	P1 vs P3: 0.40
Entitlement/Grandiosity	13.13 (0.22)	12.48 (0.85)	13.39 (0.41)	0.95^ns^	—	—
Insufficient Self-Control/Self-Discipline	12.41 (0.23)	14.20 (1.03)	13.85 (0.47)	9.19 (<.05)	P1 < P3	P1 vs P3: 0.28
Subjugation	9.60 (0.21)	12.84 (1.09)	11.91 (0.51)	23.74 (<.001)	P1 < P2, P3	P1 vs P2: 0.60 P1 vs P3: 0.44
Self-Sacrifice	15.17 (0.28)	18.78 (1.04)	16.55 (0.57)	14.19 (<.01)	P1 < P2, P3	P1 vs P2: 0.61 P1 vs P3: 0.22
Admiration-Seeking/Recognition-Seeking	12.83 (0.24)	13.35 (1.05)	13.23 (0.51)	0.65^ns^	—	—
Negativity/Pessimism	13.49 (0.30)	16.20 (1.15)	15.60 (0.65)	12.16 (<.01)	P1 < P3	P1 vs P3: 0.30
Emotional Inhibition	11.43 (0.27)	13.87 (1.00)	14.17 (0.60)	19.97 (<0.01)	P1 < P2, P3	P1 vs P2: 0.43 P1 vs P3: 0.43
Unrelenting Standards/Hypercriticalness	17.33 (0.25)	18.69 (0.72)	18.42 (0.49)	5.99^ns^	—	—
Punitiveness	11.33 (0.22)	12.31 (0.95)	12.47 (0.47)	5.09^ns^	—	—

Abbreviations: M (SE), means (standard errors); ^ns^, non-significant; P, profile; post hoc, pairwise differences with Bonferroni adjustment.

**Table 7:** Comparison of PI across latent profiles.

	**P1 (** $\mathbf{n}=\mathbf{445}$ **)** **Low Distress/High Pleasure** ***M* (SE)**	**P2 (** $\mathbf{n}=\mathbf{32}$ **)** **Deeply Affected Pleasure** ***M* (SE)**	**P3 (** $\mathbf{n}=\mathbf{135}$ **)** **High Distress/Low Pleasure** ***M* (SE)**	**Wald χ** ^**2**^ **(*P*-value)**	**Post hoc**	**Effect size**
**Psychological Inflexibility**	20.72 (0.45)	31.21 (1.73)	25.96 (0.92)	53.41 (<.001)	P1 < P3 < P2	P1 vs P2: 1.09 P1 vs P3: 0.52 P2 vs P3: 0.51

Abbreviations: M (SE), means (standard errors); ns, non-significant; P, profile; post hoc, pairwise differences with Bonferroni adjustment.

## Discussion

The profiles appear to reflect the severity of symptoms experienced. The Low Distress/High Pleasure and the High Distress/Low Pleasure profiles appear to be inversely proportional to each other, when grossly examining Figure 1. The Deeply Affected Pleasure profile shows the highest severity of symptoms, with Sexual Pleasure being the indicator that best discriminates this profile from the others. This result highlights that pleasure and distress may not always be portrayed as counterparts. The data suggesting the existence of a profile whose sexual pleasure is more compromised (when compared with the other indicators) have several implications for conceptualization, assessment, and intervention.

Current empirical evidence shows that, despite being closely related, sexual distress and sexual pleasure are independent constructs that account for negative and positive sexual experiences, respectively.^53^ Furthermore, DSF has been conceptualized as an emotionally distressing experience connected to sexual activity, leading to a reduction in the ability to experience sexual pleasure.^32^ The proper identification of individuals whose sexual pleasure appears diminished allows clinicians to intervene, even in the absence of accentuated sexual distress, to enhance positive sexual experiences in these patients.

### Profiles and EMS

The Low Distress/High Pleasure profile consistently scores lower means across all 18 EMS. These results are consistent with the fact that this cluster represents a relatively healthy group of people in terms of the indicators used to evaluate sexual dysfunction. They are also consistent with the theoretical foundation of the construct of EMS^8^ as important indicators of Sexual Health.^7^

Certain EMS in the High Distress/Low Pleasure profile show a significant increase from the Low Distress/High Pleasure profile (Table 6). The domain of Disconnection and Rejection appears to be the most affected schema domain in this cluster, ie, the creation of safe and fulfilling attachment to others is impaired.^8^ These findings are consistent with current knowledge about the association between interpersonal factors and sexual health.^7^^,^^28^ The domain of Impaired Autonomy and Performance (ie, individuals have difficulties developing a sense of independence and competence) seems to be affected as well, notably the schemas of Practical Incompetence/Dependence and Failure to Achieve, which is in line with previous research, where this domain (when controlled for sociodemographic variables and psychopathology) has been associated with impaired sexual function in both men and women.^25^^,^^26^ Our results indicate that Practical Incompetence/Dependence and Failure to Achieve may not be specific to sexual function, but also associated with distress (ie, distress related to sexual function and psychological distress) and sexual pleasure. Finally, it may also be the case that different EMS/schema domains are implicated in different dimensions of sexual dysfunction; after assessing the level and the affected components of one’s sexual dysfunction, targeting different EMS/schema domains might prove beneficial in one’s treatment.

Certain EMS (eg, Unrelenting Standards, Admiration/Recognition-Seeking) are relatively preserved when compared with the Low Distress/High Pleasure profile. These EMS partially represent the other schema domains. However, in a Turkish sample of women with a clinical diagnosis of vaginismus,^54^ these EMS were the ones that were most implicated.^55^ In another Turkish sample, Unrelenting Standards positively predicted both sexual awareness and total sexual self-confidence, while Admiration/Recognition-Seeking negatively predicted sexual awareness.^28^ Therefore, different biopsychosocial factors might be associated with impairment in different EMS/schema domains. Additionally, the individual and interpersonal perception and value attribution across different EMS might be shaped by different cultural norms and socialization patterns.^56^

Lastly, the Deeply Affected Pleasure profile scores higher means across most EMS, which is consistent with a severity-based model, putting sexual pleasure at the center of conceptualization as a dimension that contributes to higher clinical severity. When comparing with the profile of low severity, most EMS that were increased in the High Distress/Low Pleasure profile are also elevated here (with greater effect sizes), which is also consistent with a severity-based model. However, Emotional Deprivation is the early maladaptive schema that seems to distinguish this cluster from the High Distress/Low Pleasure profile. People with this early maladaptive schema expect that their desires for emotional support will not be met by others.^8^ To our knowledge, this is the first study that emphasizes the Emotional Deprivation schema as a potential factor impairing sexual pleasure. This is in line with evidence that suggests that the Emotional Deprivation schema is a negative predictor of sexual self-confidence and sexual adjustment,^28^ concepts that are linked to the experience of sexual pleasure.^57^ Outside ST, similar constructs (eg, Emotional Neglect, Childhood Maltreatment) have been implicated in the experience of sexual pleasure. A 10-year longitudinal study with a sample of mid-life adults identified that childhood emotional neglect (defined as “persistent and/or severe denial of the child’s emotional needs for love, encouragement, sense of belonging, and support”) was negatively associated with sexual pleasure in women, but no associations were found in men.^58^ Nevertheless, the sociodemographic characteristics for the Deeply Affected Pleasure profile do not allow us to conclude if these associations are gender based, as gender distributions do not statistically significantly differ across the three profiles. Taken together, these results suggest that emotionally deprived people tend not to pursue their own pleasure, leading to other adverse sexual outcomes (high distress). Therefore, screening and treatment (through ST) of individuals with high scores for the Emotional Deprivation schema might be useful in settings where diminished sexual pleasure is the most important presentation of their distress. Also, the participants of this profile tend to be older than those of the other 2 profiles (Table 5). This fact leads us to believe that these profiles are not solely presented in terms of severity and that the qualitative characteristics of the participants in the Deeply Affected Pleasure cluster might contribute to these findings. The paths through sexual pleasure become compromised throughout life, and its clinical meaning needs to be better understood in relation to EMS and beliefs about aging.^59^^,^^60^

### Profiles and PI

PI severity varies between profiles, with significant differences across them. It appears that greater inflexibility is associated with sexual dysfunction. These findings are consistent with the theoretical model where PI acts as a transdiagnostic process in overall psychological distress.^21^ However, this indicator shows no significant differences between the High Distress/Low Pleasure and Deeply Affected Pleasure profiles. Our study is the first study to assess the role of PI as a potential factor associated with diminished sexual pleasure and suggests it may have a role as a vulnerability factor. These results suggest that PI might be a potential therapeutic target for people experiencing sexual difficulties across different severity profiles. However, as stated, the profiles are likely to be heterogenous, and caution should be taken when examining associations between indicators (ie, sexual pleasure) and outcomes (ie, PI). Additionally, integrating certain psychological (eg, body image) and interpersonal (eg, relationship satisfaction) factors implicated in sexual health^7^ might prove useful in understanding how PI affects sexual pleasure.

### Profiles, PI, and EMS

To date, no studies have examined the role of PI in mediation or moderation models that explore the relationship between EMS and sexual dysfunction. However, much like the model presented by Fischer et al.,^19^ we can hypothesize that PI may contribute to the maintenance of EMS in individuals presenting with sexual dysfunction (as per the indicators used). Specifically, patients presenting with heightened Emotional Deprivation–related patterns of cognition may seek (paradoxically) less fulfilling sexual relationships, and PI may contribute to the resistance to change negative sexual experiences.

### Implications

This study shows three profiles that seem to be only partially representative of the severity of the symptoms experienced as measured by the indicators used in our analysis, stressing the need to better define the role that sexual pleasure may play in clinical conceptualization, evaluation, and intervention across different age frames, namely, the need to develop intervention protocols aimed at enhancing pleasure as a primary outcome.

In this context, the institution of interventions based on ACT or ST may be particularly relevant, given their emphasis on the transdiagnostic psychological processes associated with distressing sexual experiences that were found in the current study. ACT aims to reduce PI and promote engagement in value-consistent behaviours despite distressing internal experiences, whereas ST targets the EMS that underlie persistent emotional and behavioral difficulties. On another note, in line with Fischer et al.^19^ findings, integrating ACT and ST may be particularly beneficial in reducing inflexibility that may be (partially) responsible for the phenotypical manifestations of EMS on distressing experiences related to sexual function. This inclusion would mean that intervention in clinical sexology would necessarily take a more comprehensive approach, considering overall psychological assessment and intervention.

### Limitations

Firstly, our sample is composed mostly of women with a high educational level. Caution should be taken when examining the clinical importance of these profiles in different demographic contexts, as they may be a result of the characteristics of our sample, rather than specific clustering of isolated sexual difficulties. The results regarding EMS and PI differences in our profiles should be interpreted with this notion to consider this important limitation of our sample. More research is needed to clarify the stability of these profiles in other relevant sociocultural and demographic contexts.

The nature of this study (cross-sectional) does not allow us to infer causality in our findings, and the external validity is limited by the use of a convenience sample. Gender, sexual orientation, and relational configuration diversity were not included due to under-representativeness in our sample. Future studies including non-binary people and examining the role of sexual orientation and relational configuration diversity in the profiles are warranted.

Furthermore, relational factors (eg, relationship satisfaction)^7^ are important contributors to sexual dysfunction, but their role in this study was not explored. Thus, the extent to which relationship factor may be involved in maximizing or minimizing the effects of certain EMS on sexual outcomes remains to be explored. The role of physiological (eg, menopause, andropause) and clinical (eg, chronic disease) stressors were also not included, and knowledge should be expanded to account for these. On another note, this study is limited by the absence of a formally diagnosed clinical sample, and results that may apply to the community might not be held in clinical settings. However, our results are important as they emphasize the need to explore diminished sexual pleasure as a clinically relevant construct. Additional research assessing factors related to sexual outcomes that may delay help-seeking behaviors in these profiles is warranted.

## Conclusion

Our results suggest the existence of 3 clinically relevant profiles, 1 of them characterized by severely diminished sexual pleasure, which also exhibited qualitatively distinct characteristics, suggesting that the profiles may not be totally explained by a severity-based model. Future studies examining these characteristics are warranted. Specific EMS, such as Emotional Deprivation, and PI are both implicated in the different profiles of DSF, sexual pleasure, and psychological distress. Targeting these transdiagnostic processes in psychotherapeutic settings may prove useful in patients presenting with heightened sexual distress and diminished sexual pleasure, in the context of sexual difficulties and dysfunction also potentially contributing for overall well-being.

## References

[1] American Psychiatric Association . Diagnostic and Statistical Manual of Mental Disorders. American Psychiatric Association Publishing; 2022. 10.1176/appi.books.9780890425787

[2] Dewitte M, Borg C. Rethinking sexual pleasure in research, health care and society. Nat Rev Urol. 2026;23:333–349. 10.1038/s41585-025-01113-8 41491266

[3] Hatzichristou D, Rosen RC, Derogatis LR, et al. Recommendations for the clinical evaluation of men and women with sexual dysfunction. J Sex Med. 2010;7(1 PART 2):337–348. 10.1111/j.1743-6109.2009.01619.x 20092443

[4] Lafortune D, Girard M, Dussault É, et al. Who seeks sex therapy? Sexual dysfunction prevalence and correlates, and help-seeking among clinical and community samples. PLoS One. 2023;18(3):e0282618. 10.1371/journal.pone.0282618 PMC9987801 36877709

[5] Herder T, Spoelstra SK, Peters AWM, Knegtering H. Sexual dysfunction related to psychiatric disorders: a systematic review. J Sex Med. 2023;20(7):965–976. 10.1093/jsxmed/qdad074 37279603

[6] Abassi B, Fekih-Romdhane F, Baccar F, Cheour M, Sana E, Damak R. The impact of psychotropics on sexuality: a literature review. Eur Psychiatry. 2024;67(S1):S769–S769. 10.1192/j.eurpsy.2024.1600

[7] Brotto LA, Atallah S, Carvalho J, et al. Psychological and interpersonal dimensions of sexual function and dysfunction: recommendations from the fifth international consultation on sexual medicine (ICSM 2024). Sex Med Rev. 2025;13(2):118–143. 10.1093/sxmrev/qeae073 39786497

[8] Young JE, Klosko JS. Weishaar ME. A Practitioner’s Guide: Schema Therapy; 2003.

[9] Young J . Cognitive Therapy for Personality Disorders: A Schema-Focused Approach. Professional Resource Exchange Inc; 1990.

[10] Young J . Cognitive Therapy for Personality Disorders: A Schema-Focused Approach. Rev. ed. Professional Resources Press; 1999.

[11] Karantzas GC, Younan R, Pilkington PD. The associations between early maladaptive schemas and adult attachment styles: a meta-analysis. Clin Psychol Sci Pract. 2023;30(1):1–20. 10.1037/cps0000108

[12] Bishop A, Younan R, Low J, Pilkington P. Early maladaptive schemas and depression in adulthood: a systematic review and meta-analysis. Clin Psychol Psychother. 2022; 29(1):111–130. 10.1002/cpp.2630 34131990

[13] Tariq A, Reid C, Chan SWY. A meta-analysis of the relationship between early maladaptive schemas and depression in adolescence and young adulthood. Psychol Med. 2021;51(8):1233–1248. 10.1017/S0033291721001458 34109934

[14] Bär A, Bär H, Rijkeboer M, Lobbestael J. Early maladaptive schemas and schema modes in clinical disorders: a systematic review. Psychol Psychother. 2023;96(3):716–747. 10.1111/papt.12465 37026578

[15] Thimm JC, Chang M. Early maladaptive schemas and mental disorders in adulthood: A systematic review and meta-analysis. Int J Cogn Ther. 2022;15(4):371-413. https://doi:10.1007/s41811-022-00149-7

[16] Tariq A, Quayle E, Lawrie SM, Reid C, Chan SWY. Relationship between early maladaptive schemas and anxiety in adolescence and young adulthood: a systematic review and meta-analysis. J Affect Disord. 2021;295:1462–1473. 10.1016/j.jad.2021.09.031 34563389

[17] Kopf-Beck J, Müller CL, Tamm J, et al. Effectiveness of schema therapy versus cognitive behavioral therapy versus supportive therapy for depression in inpatient and day clinic settings: a randomized clinical trial. Psychother Psychosom. 2024;93(1):24–35. 10.1159/000535492 38176391 PMC10880804

[18] Peeters N, van Passel B, Krans J. The effectiveness of schema therapy for patients with anxiety disorders, OCD, or PTSD: a systematic review and research agenda. Br J Clin Psychol. 2022;61(3):579–597. 10.1111/bjc.12324 34296767 PMC9544733

[19] Fischer TD, Smout MF, Delfabbro PH. The relationship between psychological flexibility, early maladaptive schemas, perceived parenting and psychopathology. J Contextual Behav Sci. 2016;5(3):169–177. 10.1016/j.jcbs.2016.06.002

[20] Hayes SC, Strosahl K, Wilson KG. Acceptance and Commitment Therapy: The Process and Practice of Mindful Change. The Guilford Press; 2016.

[21] Levin ME, MacLane C, Daflos S, et al. Examining psychological inflexibility as a transdiagnostic process across psychological disorders. J Contextual Behav Sci. 2013;3(3):155–163. 10.1016/j.jcbs.2014.06.003 PMC5650239 29057212

[22] Gloster AT, Walder N, Levin ME, Twohig MP, Karekla M. The empirical status of acceptance and commitment therapy: a review of meta-analyses. J Contextual Behav Sci. 2020;18:181–192. 10.1016/j.jcbs.2020.09.009

[23] Zhao B, Wang Q, Wang L, et al. Effect of acceptance and commitment therapy for depressive disorders: a meta-analysis. Ann General Psychiatry. 2023;22(1):34. 10.1186/s12991-023-00462-1 PMC10486021 37679716

[24] Forbes MK, Schniering CA. Are sexual problems a form of internalizing psychopathology? A structural equation modeling analysis. Arch Sex Behav. 2013;42(1):23–34. 10.1007/s10508-012-9948-0 22562617

[25] Quinta Gomes AL, Nobre P. Early maladaptive schemas and sexual dysfunction in men. *Arch Sex Behav*. 2012;41(1):311–20. 10.1007/s10508-011-9853-y 21975922

[26] Oliveira C, Nobre PJ. Cognitive structures in women with sexual dysfunction: the role of early maladaptive schemas. J Sex Med. 2013;10(7):1755–1763. 10.1111/j.1743-6109.2012.02737.x 22524501

[27] Nobre PJ . Psychological determinants of erectile dysfunction: testing a cognitive–emotional model. J Sex Med. 2010;7(4_Part_1):1429–1437. 10.1111/j.1743-6109.2009.01656.x 20059651

[28] Körük S, Dok B, Vapurlu S, Başterzi M. Sexuality under the influence of the past: early maladaptive schemas and sexual life. J Sex Med. 2026;23(1):1–2. 10.1093/jsxmed/qdaf346 41370256

[29] Saito J, Kumano H, Ghazizadeh M, Shimokawa C, Tanemura H. Differences in psychological inflexibility among men with erectile dysfunction younger and older than 40 years: web-based cross-sectional study. JMIR Form Res. 2024;8:e45998. 10.2196/45998 38170587 PMC10794957

[30] Macri JA, Rogge RD. Examining domains of psychological flexibility and inflexibility as treatment mechanisms in acceptance and commitment therapy: a comprehensive systematic and meta-analytic review. Clin Psychol Rev. 2024;110:102432. 10.1016/j.cpr.2024.102432 38615492

[31] Noreen M, Jegatehsan AJ. The rising importance of psychological inflexibility: concepts, relationships, and future research trends: a systematic review. IOSR J Humanit Soc Sci. 2025;30(6):19–29. 10.9790/0837-3006071929

[32] Raposo CF, Nobre PJ, Manão AA, Pascoal PM. Understanding sexual distress related to sexual function (SDRSF): a preliminary framework based on a qualitative study with clinical sexologists. Int J Clin Health Psychol. 2024;24(3):100473. 10.1016/j.ijchp.2024.100473 PMC467054 39021678

[33] Finch WH, Bronk KC. Conducting confirmatory latent class analysis using M *plus*. Struct Equ Modeling. 2011;18(1): 132–151. 10.1080/10705511.2011.532732

[34] Dove NL, Wiederman MW. Cognitive distraction and women’s sexual functioning. J Sex Marital Ther. 2000;26(1):67–78. 10.1080/009262300278650 10693117

[35] Young J . The Young Schema Inventory, Standardized Items, 3rd Version. Schema Therapy Institute: New York. Published online; 2005.

[36] Rijo D . In: Gonçalves M, Simões MR, Almeida L eds. O questionário de Esquemas de Young (YSQ-S3). PACTOR; 2017: 159–173.

[37] Kessler RC, Andrews G, Colpe LJ, et al. Short screening scales to monitor population prevalences and trends in non-specific psychological distress. Psychol Med. 2002;32(6):959–976. 10.1017/S0033291702006074 12214795

[38] Pereira A, Oliveira CA, Bártolo A, Monteiro S, Vagos P, Jardim J. Reliability and factor structure of the 10-item kessler psychological distress scale (k10) among Portuguese adults. Ciênc Saúde Colet. 2019;24(3):729–736. 10.1590/1413-81232018243.06322017 30892495

[39] Bond F, Hayes S, Baer R, et al. Preliminary psychometric properties of the acceptance and action questionnaire-II: a revised measure of psychological inflexibility and experiential avoidance. Behav Ther. 2011;42(4):676–688. 10.1016/j.beth.2011.03.007 22035996

[40] Pinto-Gouveia J, Gregório S, Dinis A, Xavier A. Experiential Avoidance in Clinical and Non-Clinical Samples: AAQ-II Portuguese Version. *International Journal of Psychology and Psychological Therapy*, 2012;12(2):139–156.

[41] Mitchell KR, Gurney K, McAloney-Kocaman K, Kiddy C, Parkes A. The Sexual Function Evaluation Questionnaire (SFEQ) to evaluate effectiveness of treatment for sexual difficulties: development and validation in a clinical sample. J Sex Res. 2022;59(4):426–434. 10.1080/00224499.2021.1986800 34781800 PMC7616988

[42] Pascoal PM, Sanchez D, Fonseca Raposo C, Pechorro P. The sexual pleasure scale. J Sex Med. 2017;14(Supplement_4b):e308–e308. 10.1016/j.jsxm.2017.04.480 27555510

[43] Cornwall A, Jewkes R. What is participatory research? Soc Sci Med. 1995;41(12):1667–1676. 10.1016/0277-9536(95)00127-S 8746866

[44] Terry G, Braun V. Short but often sweet: The surprising potential of qualitative survey methods. In: Braun V, Clarke V, Gray D eds. Collecting Qualitative Data: A Practical Guide to Textual, Media and Virtual Techniques. Cambridge University Press; 2017:15–44.

[45] Henseler J, Ringle CM, Sarstedt M. A new criterion for assessing discriminant validity in variance-based structural equation modeling. J Acad Mark Sci. 2015;43(1):115–135. 10.1007/s11747-014-0403-8

[46] Pascoal PM, Andersson G, Fischer VJ, et al. Sexual distress with partnered face-to-face sexual activity: an exploratory qualitative study with heterosexual cis people who seek and do not seek professional help. Front Psychol. 2025;16:1553893. 10.3389/fpsyg.2025.1553893 PMC12366465 40842616

[47] Ferguson SLG, Moore EW, Hull DM. Finding latent groups in observed data: a primer on latent profile analysis in Mplus for applied researchers. Int J Behav Dev. 2020;44(5):458–468. 10.1177/0165025419881721

[48] Vuong QH . Likelihood ratio tests for model selection and non-nested hypotheses. Econometrica. 1989;57(2):307. 10.2307/1912557

[49] Lo Y, Mendell NR, Rubin DB. Testing the number of components in a normal mixture. Biometrika. 2001;88(3):767–778 http://www.jstor.org/stable/2673445

[50] Rosa PJ, Miranda IP, Pascoal PM. Uncovering latent profiles of ICT users and its relation to technostress and mental health in an adult sample: contributions of a transdiagnostic approach. Int J Hum Comput Interact. 2025;41(17):10966–10979. 10.1080/10447318.2024.2439637

[51] Bolck A, Croon M, Hagenaars J. Estimating latent structure models with categorical variables: one-step versus three-step estimators. Polit Anal. 2004;12(1):3–27. 10.1093/pan/mph001

[52] Hair JF, Black WC, Babin BJ, Anderson RE. Multivariate Data Analysis. 8th ed. Pearson; 2016.

[53] Pascoal PM, Raposo CF, Roberto MS. A transdiagnostic approach to sexual distress and sexual pleasure: a preliminary mediation study with repetitive negative thinking. Int J Environ Res Public Health. 2020;17(21):7864. 10.3390/ijerph17217864 33121015 PMC7663705

[54] Guze SB . Diagnostic and Statistical Manual of Mental Disorders, 4th ed. (DSM-IV). Am J Psychiatry. 1995;152(8):1228–1228. 10.1176/ajp.152.8.1228

[55] Dikmen S, Safak Y. Effect of early maladaptive schemas and sexual self-schemas in vaginismus. Int J Med Rev Case Rep. 2020;0:1. 10.5455/ijmrcr.effect-schemas-sexual-self-schemas-vaginismus

[56] Martin IR, Stewart SE, Tchernegovski P, Devenish BD. Cultural suitability of schema therapy: a qualitative exploration of clinician views. Aust J Psychol. 2024;76(1):2412012. 10.1080/00049530.2024.2412012 PMC12218566 40666653

[57] Chesli SR, Bostani Khalesi Z, Chenari SS. The role of sexual self-esteem, sexual desire, and sexual assertiveness in the female sexual function. Psicol Reflex Crít. 2024;37(1):21. 10.1186/s41155-024-00303-4 38861225 PMC11166604

[58] Talmon A, Uysal A, Gross JJ. Childhood maltreatment and mid-life adult sexuality: a 10-year longitudinal study. Arch Sex Behav. 2022;51(2):781–795. 10.1007/s10508-021-02030-8 34599467

[59] Fileborn B, Hinchliff S, Lyons A, et al. The importance of sex and the meaning of sex and sexual pleasure for men aged 60 and older who engage in heterosexual relationships: findings from a qualitative interview study. Arch Sex Behav. 2017;46(7):2097–2110. 10.1007/s10508-016-0918-9 28299563

[60] Bastian S, Miller LR, Carpenter LM. Heterosexual women’s pleasure trajectories: how aging helps undo gendered sexual scripts. Sociol Forum. 2025;40(3):357–372. 10.1111/socf.13050

